# 

**Published:** 1907-10

**Authors:** 


### BOOKS RECEIVED.

A Textbook of Clinical Anatomy for Students and Practitioners. By Daniel N. Eisendrath, A.B., M.D., Adjunct Professor of Surgery in the Medical Department of the University of Illinois (College of Physicians and Surgeons.) Octavo, pp. 535. Illustrated. Second Edition. Philadelphia and London: W. B. Saunders Company. (Price, $5.00.)

---

The Principles and Practice of Modern Surgery. By Roswell Park, M.D., Professor of Surgery in the University of Buffalo, Buffalo, N. Y. In one very handsome imperial octavo volume of 1072 pages, with 722 engravings and 60 full-page plates in colors and monochrome.

Lea Brothers & Co., Philadelphia and New York, 1907. (Cloth, $7.00, leather, $8.00, net prices.)

---

A Manual of Clinical Diagnosis by Microscopical and Chemical Methods. For Students, Hospital Physicians and Practitioners. By Charles E. Simon, M.D., Professor of Clinical Pathology in the Baltimore Medical College. Sixth edition, revised. Octavo, 682 pages, with 177 engravings and 24 colored plates. Lea Brothers & Co., Philadelphia and New York, 1907. (Cloth, $4.00 net.)

---

International Clinics. A Quarterly of Illustrated Clinical Lectures and especially prepared articles on Treatment, Medicine, Surgery, Neurology, Pediatrics, Obstetrics, Gynecology, Orthopedics, Pathoogy, Dermatology, Ophthalmology, Otology, Rhinology, Laryngology, Hygiene and other topics of interest to students and practitioners. Edited by W. T. Longcope, M.D. Volume III, seventeenth series. Philadelphia and London: 1907. J. B. Lippincott Co. (Cloth $2.00).

---

Progressive Medicine, Vol. IX, No. 3, September, 1907. A Quarterly Digest of Advances, Discoveries and Improvements in the Medical and Surgical Sciences. Edited by Hobart Armory Hare, M.D., Professor of Therapeutics and Materia Medica in the Jefferson Medical College of Philadelphia. Octavo, 290 pages, with illustrations. Lea Brothers & Co., Philadelphia and New York. (Per annum, in four cloth-bound volumes, $9.00; in paper binding, $6.00, carriage paid to any address).

---

Human Anatomy, including Structure and Development and Practical Considerations. Edited by George A. Piersol, M.D., Professor of Anatomy in the University of Pennsylvania. Royal octavo, pp. 2108. With 1734 illustrations, of which 1522 are original and largely from dissections by John C. Heisler, M.D., Professor of Anatomy in the Medico-Chirurgical College. Philadelphia and London: J. B. Lippincott Company. (Price, $7.50.)

---

Diseases of the Genitourinary Organs and the Kidney. By Robert H. Greene, M.D., Professor of Genitourinary Surgery at the Fordham University, New York; and Harlow Brooks, M.D., Assistant Professor of Pathology, University and Bellevue Hospital Medical School. Octavo of 536 pages, profusely illustrated. Philadelphia and London: W. B. Saunders Company, 1907. (Cloth, $5.00; half morocco, $6.50 net prices.)

---

A Textbook of Physiology for Students and Practitioners. By William H. Howell, Ph.D., M.D., Professor of Physiology in the Johns Hopkins University, Baltimore. Octavo, pp. 939. Illustrated. Second Edition. Philadelphia and London: W. B. Saunders Company. ($4.00 net.)

---

A Textbook of Practical Diagnosis. The Use of Symptoms in the Diagnosis of Disease. By Hobart Amory Hare, M.D., Professor of Therapeutics in the Jefferson Medical College of Philadelphia. Sixth edition, revised and rewritten. Octavo, 616 pages, with 203 engravings and 16 full-page plates. Lea Brothers & Co., Philadelphia and New York, 1907. (Cloth, $4.50, leather, $5.50, net prices.)

---

Insanity and Allied Neuroses. A Practical and Clinical Manual. By George H. Savage, M.D., F.R.C.P., late Physician and Superintendent of Bethlehem Royal Hospital. With the assistance of Edwin Goodall, M.D. (Lond.) Medical Superintendent of the Cardiff City Hospital for Mental Diseases. 12 mo. pp. 638. Illustrated. Fourth Edition. Chicago: W. T. Keener & Co. (Price, $2.75.)

Hospital Corps Textbook. Fourth Brigade, N.G., N.Y. Edited by Lieutenant Colonel A. Smith, M.D., Surgeon, Fourth Brigade, N.G., N.Y.

Third Annual Report of the Henry Phipps Institute for the Study, Treatment and Prevention of Tuberculosis. February 1, 1905, to February 1, 1906. Edited by Joseph Walsh, A.M., M.B.

